# Being well? A meta-ethnography of older patients and their relatives’ descriptions of suffering and well-being in the transition from hospital to home

**DOI:** 10.1186/s12913-023-09039-w

**Published:** 2023-02-06

**Authors:** Aline Dragosits, Bente Martinsen, Ann Hemingway, Annelise Norlyk

**Affiliations:** 1 Department of Public Health, Faculty of Health, Aarhus University, Bartholins Allé 2, 3, 8000 Aarhus C, Denmark; 2 Department of Medical Science & Public Health, Bournemouth University, BGB, Bournemouth, Dorset UK; 3 Department of Health and Nursing Science, Faculty of Health and Sport Sciences, Agder University, Grimstad, Norway

**Keywords:** Older patients, Relatives, Suffering, Well-being, Transition, Meta-ethnography, Phenomenology

## Abstract

**Background:**

As the average length of hospital stay decreases, more and more older patients will need support during and after the hospital transition, which will mainly be provided by their relatives. Studies highlight the enormous effect such a transition has on the lives of older patients and their relatives. However, research is lacking regarding in-depth understanding of the complexities and the notions of suffering and well-being the older patients and their relatives describe in the transition from hospital to home. Therefore, this study aims to examine the description of suffering and well-being on a deeper, existential level by drawing on existing phenomenological research.

**Methods:**

In order to synthesize and reinterpret primary findings, we used the seven-step method for meta-ethnography. Following specific inclusion criteria and focusing on empirical phenomenological studies about older patients and their relatives experiences of hospital to home transitions, a systematic literature search was conducted. Data from ten studies have been analyzed.

**Results:**

Our analysis identified three intertwined themes: i) ‘Being excluded vs. being included in the transition process’, ii) ‘Being a team: a call for support and a call to support’ and iii) ‘Riding an emotional rollercoaster’. The last theme was unfolded by the two subthemes ‘Taking on the new role as a caregiver: oscillating between struggling and accepting’ and ‘Getting back to normal: oscillating between uncertainty and hope’. Within those themes, older patients and their relatives described rather similar than contradictory aspects.

**Conclusions:**

This study offers insights into the tension between existential suffering and well-being described by the older patients and their relatives during the transition from hospital to home. Especially, the description of well-being in all its nuances which, if achieved, enables older patients and their relatives to identify with the situation and to move forward, this process can then be supported by the health care professionals. However, there is still lack of knowledge with regards to a deeper understanding of existential well-being in this process. Given the increasing tendency towards early hospital discharges, the findings underpin the need to further investigate the experiences of well-being in this process.

## Background

This paper focuses on existential suffering and well-being in the transition from hospital to home as described by older patients and their relatives in earlier research, using a meta-ethnographical approach.

Due to several challenges including demographic changes and technical developments as well as rising life expectancy, the demands on health care systems all over the world are exponentially rising [1]. When it comes to transition from hospital to home care, health systems often fall short in cross-disciplinary coordination because the focus still lies on acute rather than episodic care [2]. However, effective continuity of care is essential to ensure well-being as well as to avoid unnecessary early readmissions after hospital discharge [3, 4]. Further, the demand for support, mainly through family members, may increase even more in the transition from hospital to home due to the decrease of the average length of hospital stay over the last two decades [5]. This is especially the case for older patients (65 years and older), who often suffer from chronic conditions and need medical and social support [6]. As they often leave the hospital with ongoing care needs, the transition is strictly speaking a transfer from hospital to home care [7]. From a caring perspective, a safe and healthy transition is essential to support the older patient and their relatives with challenges that may occur during this process [8].

Former research focusing on older patients’ and their relatives’ experiences regarding the hospital to home transition indicates that it has severe impact on the lives of both older patients and their close relatives. Older patients experience multiple struggles during the transition from hospital to home, such as unexpected or early discharge [9], lack of information as well as lack of preparedness for their homecoming [9–11]. Furthermore, feelings of discontinuity of care are reported, such as struggles with the cooperation between primary and secondary care services [6, 9, 10], inclusion of several caregivers [10], as well as lack of access to follow-up services [6].

Studies also indicate that relatives have an essential role in supporting older patients during and after their hospital stay [6, 10]. The support may create a feeling of security, especially when it comes to the organization of the discharge, others taking over care, and specific responsibilities like drug management [6]. This emphasizes that a supportive network highly influences the well-being of older patients after discharge. However, research has also documented that the relatives’ daily lives are often highly affected by a transition from hospital to home care [5]. These changes in the daily routine may include giving up or decreasing their job in order to be able to support the older patients [5], difficulties with taking over care responsibilities due to their own health status [6], and feelings of being alone with the organization of and transportation to follow-up services [11]. Similarly to the issues shared by the older people, research on relatives’ experiences with the transition process also shows that an unexpected and poorly communicated discharge may contribute to their suffering, whereas being prepared for as well as being involved in the discharge process may contribute to their well-being.

Accordingly, the studies above point towards the possibility to identify the potential positive influence the transition process has on the well-being of older patients and their relatives. However, the majority of the studies primarily investigated older patients and their relatives’ experiences with the transition from hospital to home in general. While some of them focused on the perspectives of the older patients or their caring relatives, only a few combined both. Furthermore, little attention has been given toward gaining a deeper understanding of how suffering and well-being — as well as their interplay — may affect both, older people and their relatives in the transition from hospital to home.

Galvin and Todres [12] have investigated the concepts of suffering and well-being as seen from an existential phenomenological perspective. Within their humanizing framework for care, they point to the strong relation between suffering and well-being and the importance of understanding both. Whereas well-being should direct care, suffering ‘provides a human capacity for care’ [p.98] [12]. Accordingly, the in-depth understanding of suffering and well-being is complex; however, it is an essential guide for care as it is a major driver towards lifeworld led-care, which takes patients and relatives’ experiences into account [13].

Consequently, there is a need to systematically scrutinize existing studies focused on the phenomenon of hospital to home transition to identify older patients and their carers/relatives’ descriptions of what contributes to suffering and well-being respectively in the transition from hospital to home. Therefore, the aim of this study is to examine phenomenological empirical studies that can give a deeper understanding of older patients and their carers/relatives’ experiences of suffering and well-being in relation to transition from hospital to home. A meta-ethnographical approach has been followed as it seeks to interpret and translate the findings of primary research into each other rather than aggregate them [14].

## Methods

Synthesizing qualitative studies aims to get a deeper understanding of a defined research interest by reinterpreting existing qualitative research, rather than aggregating knowledge. In order to do so, there are different types of synthesis as numeric, narrative or interpretive [15]. Meta-ethnography is a seven steps interpretive method for synthesizing qualitative research with the aim to create a line of argument either to move research forward or to avoid wasted resources [14]. One characteristic that differentiates the meta-ethnographic approach from other qualitative approaches is that the interpretations of the primary research are considered as data throughout the analysis process to deepen the understanding of a phenomenon [15]. We followed the practical guide about how to use meta-ethnography for literature synthesis by Sattar et al. [16] and the eMERGe meta-ethnography reporting guidance by France et al. [17].

### Phase 1: getting started

According to Noblit and Hare [14], the first phase of a meta-ethnography is to identify the intellectual interest, which we defined as the synthesis of the existing primary data about the experiences of older people and their relatives with regards to the transition from hospital to home. The specific focus lies in developing in-depth understanding of their descriptions of suffering and well-being throughout this process and therefore, the meta-ethnographical approach has been deemed as the most appropriate method. After defining the research interest, a literature search has been conducted by the first author.

### Phase 2: deciding what is relevant

In phase 2 the search strategy has been defined. The PEO (Population, Exposure, Outcome) framework has been used to identify keywords and search terms (see Table 1) [18]. The population of interest included older patients and their relatives. A relative can be operationalized as any person who supports the older patient during and after discharge (for instance, a family member, neighbor, or friend). The transition from hospital to home was the exposure of the search. The outcome focused on the description of suffering and well-being as experienced in the lifeworld [19] of the older patients and their relatives. Phenomenology in its essence is interested in how a particular phenomenon is experienced in the everyday life, the lifeworld, of human beings [19]. Therefore, we decided to focus on studies which followed a phenomenological approach.

**Table 1:** PEO elements and keywords

Population	Exposure	Outcome	Study type
older patient	discharge	experience	phenomenolog*
older people	hospital discharge	well-being	hermeneutic*
elderly	early discharge	well being	lifeworld
older adult*	discharge process	wellbeing	
relative*	transition	suffering	
partner	care transition	perspective*	
spouse	hospital to home	view*	
next of kin	hospital-to-home transition	need*	
next-of-kin		description*	
significant other			
informal caregiver			

*was used as a truncation

Keywords and search terms for every PEO element were identified. With the support of a research librarian, the chosen terms were discussed, and a research strategy comprising truncations, abbreviations, and Boolean operators was developed. The systematic literature search was conducted between September and October 2020 and was updated in October 2021 with no additional findings. The databases used to search for primary data included PubMed, Embase, CINAHL, APA PsycInfo, and Scopus. The database search was supplemented by screening the reference list of the included papers.

Eligibility criteria guided the screening process. Inclusion criteria comprised studies which followed a phenomenological approach focusing on the experiences of older patients and/or their relatives on transition from hospital to home. The search was limited to studies published in English, German or Danish within the last 10 years. Our research interest was to focus on the older patients and their carers/relatives’ experiences of suffering and well-being in relation to transition from hospital to home. Therefore, studies investigating the perspectives of older patients and/or their relatives on transition from hospital to other care institutions (e.g. nursing homes) were excluded. Moreover articles examining perspectives other than those of older patients and/or their relatives as well as studies that did not follow a phenomenological approach were also excluded. Finally, studies were included if the majority of the participants met the definition of older patients [1].

Following the specified inclusion and exclusion criteria, the first author and a second reviewer screened the abstracts independently. In the case of a disagreement, the inclusion was discussed, and a decision was reached together. The first author performed the data extraction. Every step was discussed with and approved by all authors.

**Fig. 1:**
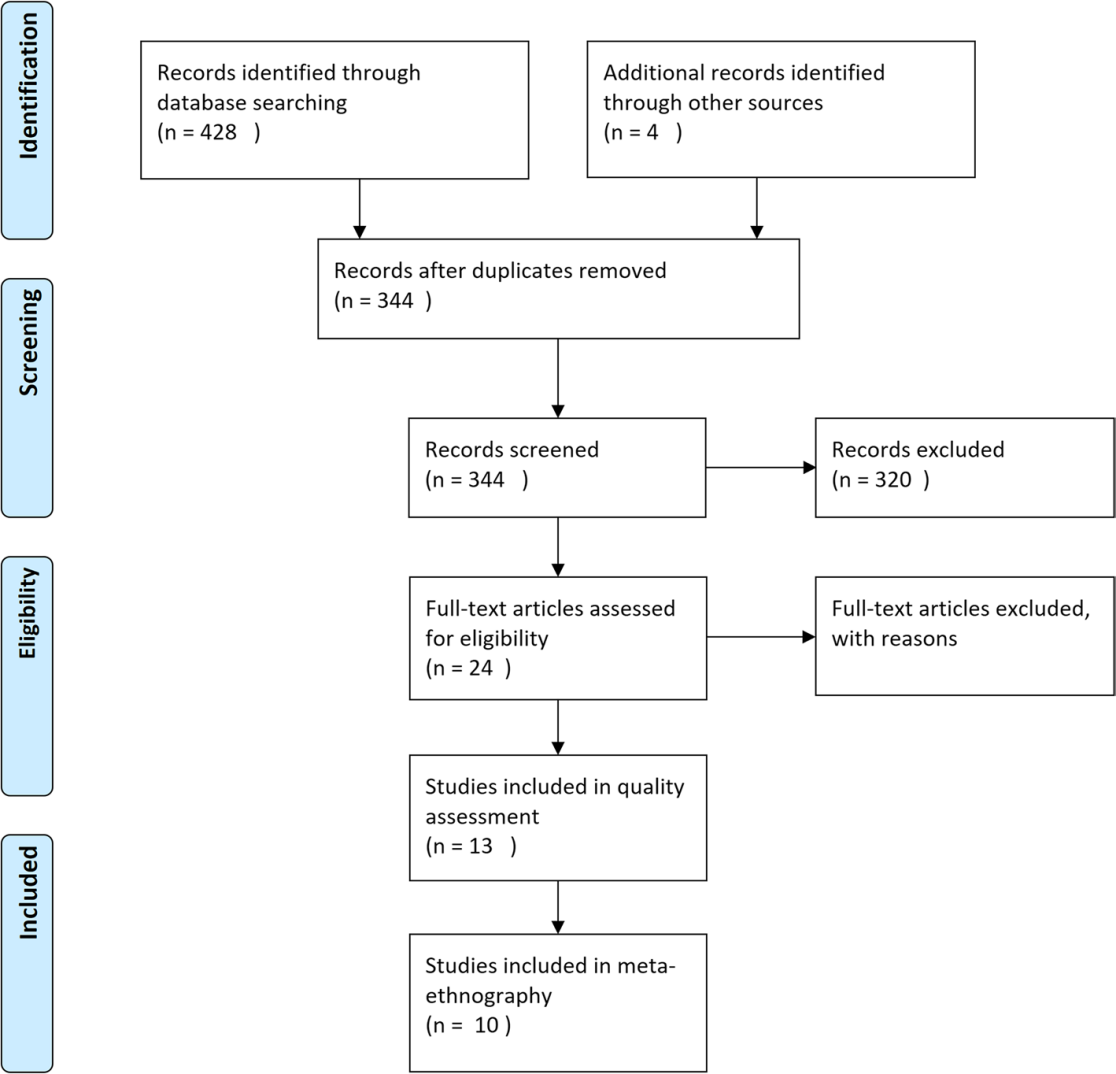
PRISMA flow-diagram

Figure 1 illustrates the selection process summarized in a Preferred Reporting Item for Systematic reviews and Meta-Analysis (PRISMA flow diagram) [20].

The systematic database literature search identified 428 articles. Four more articles were derived through grey literature and a manual search for additional studies in the reference lists of eligible studies. After removing duplicates, 344 titles and abstracts were screened by following the defined exclusion criteria. This led to 24 articles, which were read in full text and then assessed against the inclusion criteria. Following the screening process, 13 articles remained, and were subjected to an assessment by using the Critical Appraisal Skills Program (CASP) checklist (Critical Appraisal Skills Programme 2018). The Cochrane guidelines and the World Health Organisation recommend the use of the CASP checklist for qualitative evidence synthesis [17]. Originally, there was no assessment process outlined in Noblit and Hare’s [14] seven steps process. Nevertheless, Campbell et al. [15] recommend following an appraisal process in order to become more familiar with the studies. Therefore, the CASP checklist was used to ensure that the studies were relevant to the phenomenon of interest rather than merely as a quality assessment [16, 21]. In this process, three studies were removed. In the final stage, ten studies that met the aforementioned inclusion criteria were identified.

### Phase 3: Reading the studies

In order to become familiar with the findings and to start the synthesis, the articles were read and re-read [14, 21]. The aim of this process was to identify interpretative metaphors, as described by Noblit et al. [14]. Interpretative metaphors are key concepts and can be understood as base for the data synthesis [15]. Contextual information was extracted to a table. Following the aim of our meta-ethnography, relevant codes from the primary study were extracted by using NViVo. Thereby, first-direct quotes- and second order constructs- authors interpretations- of the included studies were considered. Metaphors which emerged from the analysis of the raw data were noted down.

Table 2 provides an overview of the study characteristics. The studies were published between 2010 and 2020. Most of the studies were conducted in Europe (Denmark, Italy, Norway, and Spain). Two studies were conducted outside Europe— one in the United States and one in New Zealand, respectively. While some studies focused on a specific condition (e.g., colon cancer, hip-fracture), others had a more general perspective. Furthermore, the included studies followed different phenomenological approaches. Eight studies defined themselves as descriptive and hermeneutic phenomenological, one as thematic analysis with a phenomenological perspective and one as an interpretative phenomenological analysis. Table 2 provides an overview of the themes and example quotes which have been included in the analysis.

**Table 2:** Summary of phenomenological studies included in the meta-ethnography

Perspective	Authors/Year/Title	Aim	Informants/Country	Methodology/Analysis	Key findings/Themes	Sample quotes
Patients	Hvalvik et al. (2015) [22] *The transition from hospital to home: older people’s experiences*	The study aimed to describe and illuminate the lived experiences of older home residents with continuing care needs during the transition from hospital to home	*Informants*: Seven patients aged 67 years or more (between 70 and 80 years) (2 m / 5 f) *Country*: Norway	Individual narrative interviews Phenomenological-hermeneutic approach (Lindseth et al. 2014; Ricoeur 1976)	Two themes/four subthemes: *Relating to different systems of care* - Feeling disregarded - Being humble *Adapting to life conditions* - Being vulnerable - Coping with alterations	“I could never have managed without my daughter. She has visited me every day after I returned home from hospital!”
	Lilleheile et al. (2020) [23] *A qualitative study of old patients’ experiences of the quality of the health services in hospital and 30 days after hospitalization*	The aim of this study was to investigate the experiences of older patients (80 years and above) in hospital and shortly after hospitalisation	*Informants*: Eighteen patients (82–100 years) (3 m / 15 f) *Country*: Norway	Semi-structured interviews Thematic analyses with a phenomenological perspective (Braun and Clarke 2006)	Five themes: - Hospital stay and the person behind the diagnosis - Poor communication and coordination - Life after discharge - Relationships with next of kin - Organizational and systematic determinants	“So I thought maybe I should have some papers with me? I didn’t receive any information about anything. I found that strange too. I knew I was going home, but there was no information about any possible consequences of my disease, if there was something I should think about when I came back home, for instance, what to eat, and how to take medication and stuff. Nothing at all.”
	Perry et al. (2011) [24] *“If I didn’t have anybody, what would I have done?”: Experiences of older adults and their discharge home after lower limb orthopaedic surgery*	The study explored the perceptions of older patients who returned home after lower limb orthopaedic surgery	*Informants*: Eleven patients (66–88 years) (3 m / 8 f) *Country*: New Zealand	Semi-structured interviews Interpretative phenomenological analysis (Smith et al. 2009)	Three themes: - Lack of a shared decision on when to go home - Dependent on family to go home and to feel confident there - Trial and error rehabilitation	“If I didn’t have anybody, what would I have done?”
Relatives	Hvalvik et al. (2015) [25] *Striving to maintain a dignified life for the patient in transition: next of kin’s experiences during the transition process of an older person in transition from hospital to home*	This study aimed to examine the next of kin’s lived experiences during the hospital-home transition of an older person with ongoing care needs	*Informants*: Eleven next-of-kin of an older patient (age 67 and older) *Country*: Norway	Interviews; Phenomenological hermeneutic design (Lindseth et al. 2004, Ricoeur 1976)	Two themes/four subthemes: *Balancing vulnerability and strength* - Enduring emotional stress and frustrations - striving to maintain security and continuity *Coping with an altered everyday life* - Dealing with changes - Being in readiness	“I have to visit my father daily, so I have to adjust my own routines.” “Our family had its own shift system during the first period after mum returned home, as she was anxious all the time.”
	Mora-López et al. (2016) [26] *Analysis of the transition process among family caregivers in a hospital in the region of Catalonia in Spain*	The aim of the study was to examine the experience of family caregivers who play a major role in the hospitalisation, transition process and patient care at home	*Informants*: Six family caregivers (48–78 years) (1 m / 5 f) *Country:* Catalonia, Spain	In-depth interviews; Phenomenological approach as well as Glasers and Strauss constant comparative method (Wimpenny 2000, Giorgi 2006, Medina Moya 2005)	Seven themes: - Cultural beliefs and attitudes - Religious beliefs - Meaning of ‘take care’ - Training and knowledge - Socio-economic status - Community - Society	“At first I had to give up work, I asked for a leave of absence and I had to give it up, because I think she comes first now.”
	Nahm et al. (2010) [27] *Exploration of Informal Caregiving Following Hip Fracture*	The aim of the study was to explore the experiences and needs of informal caregivers of hip fracture patients	*Informants:* Ten informal caregivers (49–86 years) (care recipients 80–93 years) (1 m / 9 f) *Country*: United States	In-depth interviews; Descriptive and hermeneutic phenomenological approach (Van Manen 1990, Barritt 1985)	Five themes/two phases: *Caregiving Experience Consistent across different phases* - Hip fracture as a turning point toward a frailer state - Feeling tired and having experienced a lack of sleep due to demanding care-giving activities - Being frustrated with the lack of communication from the health care providers and communication loopholes during transition of care - Lack of information about care-giving activities - Specific resource needs *Phase-specific caregiving experiences*	“Um, very stressful. Um, it was just trying to juggle everything. You know, work, making sure things were taken care of with his household, my household, visiting him every day.”
	Norlyk et al. (2013) [28] *The extended arm of health professionals. Relatives’ experiences of patient’s recovery in a fast-track programme*	This study examined the lived experiences of being a close relative to a colon cancer patient	*Informants*: Twelve relatives (45–75 years) (3 m / 9 f) *Country*: Denmark	In-depth interviews; Descriptive phenomenological approach, Reflective Lifeworld Research (Dahlberg et al. 2008)	Four themes: - The extended arm of the professionals - Alertness directed at the physical restoration of the patient - Organization of a healing environment - Existential pressure	“For me, it has not really been all that difficult because there were these guidelines you were supposed to follow. Those guidelines helped us on the way and we’ve done everything by the book … we really have.”
	Plank A. et al. (2012) [29] *Becoming a caregiver: new family carers’ experience during the transition from hospital to home*	To investigate the experiences of hospital-home transitions of new informal caregivers	*Informants*: Eight were interviewed individually, ten of them were part of a focus group (32–80 years) (3 m / 15 f) Care receivers (46–86 years) (12 m / 6 f) *Country*: Italy	In-depth and focus group interview Phenomenological approach (Colaizzi 1978, Giorgi & Giorgi 2003, Mortari 2008)	Core concept: - Being responsible for everything Three themes: - Reflections on the newly acquired role as family caregiver - Reflections on the recipient’s condition - Reflections on the support required to carry out the role of carer	“He is a fighter. Maybe it’s because of this that I feel more comfortable than others. Because of his strength of will! And this is an important benefit for me.”
Patients and relatives	Jensen et al. (2017) [30] *“If only I had known”: a qualitative study investigating a treatment of patients with a hip fracture with short time stay in hospital*	The study aimed to describe the experiences of hip fracture patients with regard to self-care after short time stay in hospital	*Informants*: amongst others ten patients (67–92 years) (2 m / 8 f) and four relatives (2 m / 2 f) *Country*: Denmark	Interviews Observations Descriptive phenomenological approach, Reflective Lifeworld Research (Dahlberg 2010, Dahlberg et al. 2008, Kvale 1996) Field observations within wards (Spradley, 1980)	Two categories/six themes: *Patient perspectives of the pathway:* - Preconceived notions - Importance of autonomy - Master in my own house - Will and zest for life *Self-care and empowerment:* - Preparing for discharge - Cross sectional collaboration	“The first week after I came home I actually got better every day.”
	Petersen et al. (2021) [31] *A challenging journey, the experience of elderly patients and their close family members after major emergency abdominal surge*ry	The study investigated the experience of patients and their caregivers with regard to major emergency abdominal surgery during hospitalisation and in the first month after discharge	*Informants*: Fifteen patients (70–90 years) (6 m / 9 f) and twenty of their family members (8 m / 12 f) *Country*: Denmark	Semi-structured interviews Phenomenological approach (Giorgi 2009)	Three themes: - Being emotionally overwhelmed - Wanting to be cared for - Finding a way back to life	“I’m sick of being stuck in my apartment on the second floor – walking from the bedroom to the kitchen. That’s how my days go by. I’m sick of it … really. But I can’t do anything else.”

*m* Male, *f* Female

The theoretically informed framework concerning an existential understanding of suffering and well-being by Galvin and Todres [12] was used as guideline for identifying patients’ and relatives’ descriptions of suffering and well-being. The core element of the humanizing framework for care is the lived experience of others within the care setting, which is grounded in lifeworld theory [12, 13]. The constituents of the individual lifeworld are five existential dimensions—temporality (e.g., the experienced time rather than clock time), spatiality (e.g., the felt space different from the quantitative measurements), intersubjectivity (e.g., the experience of how we are with others rather than relationships), embodiment (e.g., the felt body different from the objective body) and mood (e.g., the felt emotional attunement rather than feelings) — which form the underlying domains of suffering and well-being [12]. These two both imply one another and are intertwined [12].

### Phase 4: determining how the studies are related

Phase 4 of a meta-ethnography consists of determining how the studies are related [14]. Although, the included studies followed slightly different phenomenological approaches, they were similar concerning data collection and the setting of recruitment. Further, they were sufficiently similar in their focus on the older patients and relatives’ experiences of the transition from hospital to home. The studies did not provide detailed information about participants characteristics (e.g. gender, ethnicity, etc.), however, this information was not considered as relevant following the aim of this meta-ethnography. Accordingly, the included studies allowed for a reciprocal translation synthesis [15]. In order to determine how the included studies were related thematically, a list of metaphors identified in Phase 3 has been screened to find similarities and patterns. Furthermore, this list has been summarized to relevant categories such as concept of dependency after discharge, putting own needs aside or struggling with a lack of information for example.

### Phase 5: translating the studies into one another

Within phase 5, the first step was to translate the studies into one another by considering the interpretated categories as analogies [14]. To accomplish this, the articles were arranged according to their appraisal as well as chronologically. The metaphors from study one were compared with those from study two and so on in order to synthesize the findings and get a higher order interpretation in the form of concepts [14, 21].

### Phase 6: synthesising translations

Phase 6 of a meta-ethnography consists of ‘making a whole into something more than the parts alone imply’ (p. 28 [14]). The similarity of the included studies allowed a reciprocal synthesis in which all studies were included. Furthermore, the aim of this phase was to put the outcome of the reciprocal synthesis into a line of argument, which represented a higher order interpretation [21]. In order to reach this aim, we started to summarize the identified metaphors and list of categories and juxtaposed them with the first and second order constructs [16]. Following this process, we were able to create third order constructs in the form of themes and to reach a deeper understanding of older patients and their carers/relatives’ experiences of suffering and well-being in relation to transition from hospital to home. This led us to a line of argument synthesis, which contributed to the higher level understanding that older patients and their relatives experienced similar aspects of the same phenomenon ‘transition from hospital to home care’ rather than contradictory ones.

### Phase 7: expressing the synthesis

The following section will present the outcome of our line of argument synthesis.

## Results

During the analysis, three intertwined themes and two subthemes became apparent to understand older patients and their relatives’ in-depth description of suffering and well-being in the process from hospital stay to discharge and home care recovery:

- Being excluded vs. being included in the transition process
- Being a team: a call for support and a call to support
- Riding an emotional rollercoaster
  - ◦ Taking on a new role as a caregiver: oscillating between struggling and accepting
  - ◦ Getting back to normal: oscillating between uncertainty and hope

### Being excluded vs. being included in the transition process

The interaction with the health care professionals during the transition from hospital to home care was perceived as a crucial cornerstone by the older patients as well as their relatives. Feelings of being seen as an object and a sense of being excluded from the transition process for both the older patients and their relatives caused suffering. In line with this experience, the studies also indicated that the perception of being included in the transition process could be a source of well-being for both parties.

Being seen as an object and the feeling of being excluded in the transition process were experienced in different ways. In some instances, older patients addressed it as a feeling of being invisible rather than being informed about the discharge and transition plan, as well as potentially feeling disempowered from moving on with their lives at the current stage of their illness [22, 23]. Accordingly, this approach created suffering for both patients and relatives and especially forced older patients to take on a passive role [23]. Although many patients chose to accept their passive role by putting their trust in the health care professionals [24, 30], there was also exhaustion, insecurity, and concern due to the experienced one-way communication [23]. This was described as a lack of dialogue about the discharge or how to cope with unresolved health problems [23, 24, 30]. A ‘professional language that was not understandable’ [23], as well as no opportunity to participate in the transition process, alienated the older patients from identifying themselves with this new situation [22, 23], thus, nourishing the feeling of being seen as an object of care.

The relatives also echoed the experiences of the older patients and described those feelings of being seen as an object aligning with being excluded; for example, when they struggled with asking relevant questions due to their fears of being unable to fully comprehend the given information [28]. Furthermore, information was perceived to be hardly understandable and, when coupled with an abrupt discharge, was experienced as a loss of control and stressful by relatives [25, 31]. Additionally, the experience of being excluded was described as a feeling of being co-responsible for care but without being involved in the process by the health care professionals [24, 25, 28, 31].

To address the patients’ needs, the relatives felt responsible for effective and clear communication between the hospital and home care nurses [25]. When relatives experienced the relationship with the health care professionals as distanced [25], this responsibility was expressed as frustrating and stressful. Relatives felt as if they were removed from the care process and not taken seriously concerning their ability and desire to support the older patient during and after the discharge [26]. Furthermore, they felt that the system did not take their situation as new caregivers seriously enough [25]. Consequently, the overwhelming feelings of responsibility as well as being excluded and alone were hard to bear.

To feel involved and secure, it seemed that dialogue and information were experienced as key factors by older patients and their relatives. More specifically, this can be explained by the patients’ and relatives’ strong desire to be informed and actively involved in the transition process [23, 25, 27, 30]. Not only was this experience important for them to feel prepared for every phase of the transition process, but also for the recovery at home to occur [23, 29, 30]. Hence, a clear, individualized, proactive, and timely discussion during the discharge process, as well as information about how to deal with the home recovery, were pointed out as important factors for older patients and the relatives [24, 31]. These particular factors strongly contributed to their experience of well-being.

### Being a team: a call for support and a call to support

The interplay between a call for support and a call to support reflected the essence of being a team in the transition process. This intersubjective relation between older patients and their relatives clearly established itself to be an important aspect, with the potential of creating both suffering and well-being, as it was strongly driven by the dynamic within the family.

The abrupt changes in the lives of the older patients caused by the hospital stay led to a silent call for support due to dependency on help from their relatives, as the older patients were all of a sudden unable to deal with daily tasks [23]. This dependency could lead to suffering for both parties. For example, the awareness that the relatives needed to adapt their lives in order to assist the patients [24] created a feeling of being a burden [23, 24]. Further, patients experienced potential worries that the dependency on their relatives’ support could affect their relationship in a negative way [23]. They tried to be as low maintenance as possible, although it sometimes demanded a lot of compromise, such as overlooking when the relatives fulfilled everyday tasks in a different way than the older patients were used to [24].

The call to support could change the everyday lives of the relatives drastically as well, as the new responsibilities and resulting changes in their everyday lives could lead to suffering. The fluctuations in the older patients’ moods and perceived changes in their personalities made the relatives worry about the well-being of the care receivers [28, 29]. They were constantly in a state of inner struggle about whether they were supporting too little or too much [28]. Consequently, the added responsibilities and their new reality influenced their own well-being and could lead to suffering.

Nevertheless, it seemed that the support from the relatives is essential for older patients to feel well. For example, the support was experienced as a safety net for hospital and home care [22, 23, 31], which strongly contributed to the patients’ well-being. The assistance of relatives made them feel comfortable enough to be transferred to and cared for at home [23–25]. The feeling of not being alone at home in general, but also in case something unexpected happened, was a relief [24]. Following daily routines as a team especially supported the relatives within their new role [28]. For the relatives, a positive and co-operative attitude from the older patients also contributed to their own personal well-being [29]. It seemed that a strong relationship and a feeling of trust highly influenced the well-being of the older patients, which consequently influenced the well-being of the relatives as well.

### Riding an emotional rollercoaster

The primary research reported many emotional struggles for older patients and their relatives in identifying themselves with the situation caused by the hospital stay and transition. This can be interpreted as a ride on an emotional rollercoaster. For the relatives, this description expressed concerns oscillating between struggling with and accepting the new role as a caregiver, and for the older patients as oscillating between uncertainty and hope to get back to a life as they knew it before the hospital stay.

#### Taking on the new role as a caregiver: oscillating between struggling and accepting

The struggle with and the acceptance of the new situation after discharge was a recurring theme for the relatives, triggered by the hospital stay and transition to home. It seemed that the hospitalization of the older patients especially forced the relatives to adapt to new roles. The relatives became caregivers, and the older patients became dependent—or in some cases even more dependent than before—on support.

Relatives considered themselves as pilots who had to navigate through the chaos. Some relatives experienced exhaustion [27], insecurity [25], and frustration [31] because they felt frail and not prepared enough for being a caregiver [25, 31]. In some cases describing feelings of always being on call [25, 28]. Together with the co-responsibility for the recovery of their loved ones [25], these aspects contributed to suffering for relatives; for example, if they were not prepared for the new responsibilities [25, 27, 28]. Especially during and immediately after the transition from hospital to home, they experienced a high degree of responsibility to take over tasks for the older patients in order to ensure that the patients were able to carry on with their everyday lives [25]. These tasks included personal hygiene, managing medications [28, 29], and communication with health care professionals [29].

Relatives could be exhausted with keeping all these balls in the air. It seemed that the pressure of being responsible for many things suddenly, alongside the fear of not having enough resources, created suffering. They experienced feelings of uncertainty, stress, and being alone and lost [25, 28, 29], which led to worries, fear, doubt, and vulnerability [29]. Uncertainty about the health conditions of the patients, expectations about the patients’ behavior, as well as anxiety about how to deal with these new circumstances were the driving force behind those feelings [25, 28, 29]. This, coupled with the constant tension between their own needs and the needs of their loved ones [25, 28], caused suffering.

However, relatives also found their new role as caregivers for their loved ones an obvious obligation [25, 28, 29], and they expressed pride in this new role [25, 29]. Specifically, they indicated that they were able to identify with their new role when they felt involved in the care process and prepared for the therapeutic requirements at home [29]. Furthermore, it seemed that sleep and proper rest as a form of self-care were important factors contributing to their well-being. For instance, they experienced needing to be aware of their own needs as important and needing to take breaks in order to recharge their batteries [28].

#### Getting back to normal: oscillating between uncertainty and hope

Getting back to normal after the transition from hospital to home appeared as a central theme for the older patients. It seemed that time and bodily perceptions played an important role for the older patients in orienting themselves during and after the transition from hospital to home care.

Uncertainty about the amount of time it would take to get back to the life the older patients knew before the hospital stay [22, 24] caused suffering. This uncertainty could further be exacerbated by a lack of time to prepare for the transition, as well as uncertainty regarding future care needs [22, 23, 31]. Furthermore, the studies also described a sense of suffering caused by the uncertainty whether the physical conditions would actually allow for such a return to ‘normal’ [22, 31]. Especially immediately after the transition, older patients experienced feelings of being caught in their own bodies due to reduced physical health status [31]. This was for example experienced as not being able to participate in common daily activities, changing patients’ experiences of the home to a restricted area [23], which in turn gave rise to suffering.

Although the homecoming could be challenging, it seemed that the older patients were relieved to be at home again, which contributed to their well-being [24]. The hope that time will make it possible to get back to life as they knew before the hospital stay [24, 31] played a central role with regards to their well-being. In order to nourish this hope, it seemed important for them to be able to deal independently with everyday tasks as soon as possible [23, 24, 30]. Furthermore, preserving their self-respect and dignity were experienced as essential in order to face challenges and preserve well-being [22].

## Discussion

These findings indicate that both older patients and their relatives experience suffering and well-being on an individual level but also due to changes in their relationship during and after discharge. In line with other studies [6, 9–11], the findings drew attention to the overall experience of immense suffering, but importantly also provided a glimpse of experienced well-being. Our study provides important additional insight into former research as it became apparent that the description of suffering and well-being in the transition process from hospital to home in our findings affected the lifeworld’s of the older patients and their relatives in several ways, specifically the existential dimensions intersubjectivity and temporality.

As our results show, the experienced suffering and well-being in the transition from hospital to home care appear to be due to the constant struggle concerning security and certainty about care for the older patients and their relatives. Older patients experience insecurity about care when they do not feel included and informed by the health care professionals. They mainly experience uncertainty about care immediately after the discharge if their future care provision and plan is perceived to be vague. Relatives experience insecurity with their new responsibilities as caregivers, with uncertainty about how to care and what is needed which leads to suffering. In contrast, security and certainty that older patients will receive the care they need contribute to well-being during and immediately after the discharge. The findings show that relatives experience well-being when they feel secure in their roles and certain that they are able to fulfill their roles as caregivers with the support of the older patients and the health care professionals.

Consistent with former research [5, 9–11], the findings point to the fact that the perception of preparedness is a strong indication for the older patients and their relatives’ experiences of either suffering or well-being. Importantly, the findings of the current study provide essential additional insights on suffering and well-being with regards to inclusion and preparedness on a deeper, existential level. Well-being in this sense is about feeling safe and ready to be transferred to home, which enables a sense of possibility (mobility), whereas the certainty of and how to administer care enables a sense of settledness (dwelling) [12]. Well-being is, in its essence, a sense of homecoming (dwelling) as well as possibility (mobility). ‘When dwelling and mobility are intertwined,’ this ‘constitutes the deepest possibility of well-being’(p. 681 [32]). Following this logic, it becomes apparent how existential suffering and well-being are interrelated. ‘Suffering announces vulnerability, and well-being announces freedom’ (p. 98 [12]).

More specifically, the findings illustrate that the existential dimensions of intersubjectivity and identity strongly relate to suffering and well-being in the transition from hospital to home and highly depend on the experienced relationship with the health care professionals. On the one hand, interaction with the health care professionals contributes to the above-described insecurity and uncertainty when experienced as distant and unclear. On the other hand, security and certainty about care can be experienced when the interaction and perceived care enable identification with the new reality caused by the transition for both the older patients and the relatives. Those findings are supported by previous research [5, 9, 10], which shows that the perceived relationship with the health care professionals has a severe influence on the experienced homecoming. Our study adds to the prior literature by showing the influence this relationship has on existential suffering and well-being. According to Galvin and Todres [12], the dwelling-mobility of suffering in the identity dimension can be understood as the vulnerability causing a feeling of losing oneself and being useless, which leads to an experience of fragmentation. This alienation is strongly intertwined with the experienced isolation the interaction with the health care providers brings with it [12]. In contrast, an inclusive transition process driven by the health care professionals enables the dwelling-mobility of the older patients and relatives. It is a feeling of ‘I can’ identify with the current health condition and caring situation as well as a continuum with the dwelling aspect of ‘I am’ able to face the current health condition and caring situation due to support [12].

Moreover, our findings highlight that the existential dimension of intersubjectivity was not only limited to the relationship with the health care professionals but also between the older patients and their relatives, which was another important aspect with regards to the in-depth understanding of suffering and well-being. Importantly, the findings show that this can be further understood as an intersubjective bond between the older patients and the relatives, which unfolds itself into different nuances of dependency and interdependency. Usually, dependency and especially dependency on care are experienced on an individual level [33–35]. However, this study emphasizes the intertwined experiences and different nuances of dependency in the relationship of the older patients and their relatives. For the older patients, the dependency on care leads to suffering, especially when they experience themselves as a burden who is in dire need of support. This finding is in line with the dwelling suffering in the intersubjective dimension, according to Galvin and Todres [12], where the feeling of being an outsider dominates. In contrast, our findings show that dependency can only be experienced when there are people whom one can depend on for support. Furthermore, our study shows that this perception of support and having their relatives be there for them allows the older patients to experience well-being. This is the existential dimension of well-being, as this feeling of kinship and belonging is described in the dwelling well-being of the intersubjective dimension [12]. Relatives in our findings experienced the dependency of the older patients as a major life-altering event, which comes with new responsibilities. On the contrary, dependency for the relatives led to a sense of inner pride and emotional fulfillment, which enabled an experience of well-being. Galvin and Todres [12] describe this as the deepest form of well-being in the intersubjective dimension. It is defined as mutual complementarity and can be understood as a continuum of kinship and belonging as well as a mysterious interpersonal attraction [12]. Another nuance of dependency we uncovered in our findings is the emotional co-dependency, meaning that the experienced mood and with it suffering or well-being of the relatives are highly dependent on the mood of the older patients and vice versa. Those findings add a deeper level to previous research [5, 6], which especially pointed to the importance the support from the relatives can have for the older patients.

As shown in our study and underlined by the theory, there is a strong bond between the existential dimensions of intersubjectivity and mood. The existential dimension of mood is strongly entangled with all other lifeworld dimensions and forms them [12]. Our findings are especially in line with the dwelling dimension of mood when it comes to the experienced co-dependency. The existential dwelling and suffering in mood can be interpreted as irritation [12], when the moods of relatives are irritated by the moods of the older patients and vice versa. According to Galvin and Todres [12], the same can be found for existential well-being. When mood is experienced as peaceful, well-being is possible.

Furthermore, our findings show how the existential dimension of temporality has a major influence on suffering and well-being in the hospital to home transition for the older patients. Time, as experienced, is a crucial factor. This conclusion is also supported by Dolu et al. (2021). However, our findings contribute to a more nuanced understanding of how time is related to existential experiences of suffering and well-being. This study uncovered the notion that time was experienced with anxiety with regards to handling future care and daily demands. According to Galvin and Todres [12], this is suffering in the existential dimension temporality. Any sense of the future is blocked, and the present is uncertain. Our findings strongly point to the fact that time is related to the hope that the future will bring back life as it used to be for the older patients. This is the existential dwelling mobility of well-being in the dimension of temporality, described by Galvin and Todres [12] and can be understood as acceptance that life is not like it used to be and that there is an opportunity to find peace within the current situation.

The strength of this study lies in the intertwined description of older patients and their relatives suffering and well-being in the transition from hospital to home. By choosing a meta-ethnographical approach, it was therefore possible to reinterpret and translate the primary research findings, even though the majority of the included papers considered either the experience of older patients or their relatives. Nevertheless, there are potential limitations to this study. Focusing on the lived experiences of older patients and their relatives, only phenomenological studies have been included. As the number of studies following this approach is limited, studies have been included if the majority of participants fulfilled the defined inclusion criteria for older patients. Nearly all studies focused solely on this target group. In case of one study regarding the relatives’ experiences, the age of the older patients was not mentioned. However, considering the indication for the hospital stay, it can be assumed that the majority of the patients can be defined as old. Only one study considering the relatives perspective also included patients, which were younger than 65 years. Further the included studies didn’t provide information about whether the patients received home care or not. The above aspects might be considered as a limitation. Another limitation might be that the search was limited to studies published in English, Danish and German. Therefore, relevant studies published in another language might be missing in this meta-ethnography.

## Conclusion

This meta-ethnography of ten phenomenological studies focused on the descriptions of older patients and their relatives during the transition from hospital to home. It illustrates a tension between suffering and well-being on the existential level for both parties. Overall, our results show that the experience of security, i.e., dwelling, as well as the certainty related to future possibilities or mobility, are essential in order to form a more existential—and with it, also a more holistic picture of suffering and well-being in the transition from hospital to home. For both the older patients and the relatives, the burden of uncertainty is associated with the ego dystonic or alien situation caused by the transition from hospital and, most importantly, with the insecurity about what to expect from future life and how to adapt to the new reality. This fear of the unknown can be seen as fertile soil for existential suffering. Nevertheless, the certainty of the presence of support enables both, older patients as well as relatives to carry on. Especially dependency perceived as support between the older patients and the relative creates a sense that neither the older patients nor the relatives have to face the burden of uncertainty and insecurity by themselves. In conclusion, a bond of trust between the health care professionals and also between the older patients and the relatives positively supports the experience of well-being in all parties.

These factors identified in our current work can be supported in care practice. In order to ensure well-being in the transition process from hospital to home, it is important for health care professionals to enable a feeling of layered continuity for older patients and their relatives by supporting them to identify with the unfamiliar situation triggered by the transition. With regards to the relationship between the older patients‘ and the relatives’, they need to be engaged to reconnect to each other according to the older patients and relatives needs and possibilities.

Our findings indicate that focusing on the experience of well-being has the potential to significantly contribute to a successful transition from hospital to home. So far, little attention has been given to the existential dimension of well-being experienced by older patients and their relatives in the transition from hospital to home. Therefore, there is a need for further investigation in this important area, especially due to the current trend of early discharge.

## Data Availability

Data sharing is not applicable to this article as no new data was created or analyzed in this study.

## Abbreviations

CASP: Critical appraisal skills programme
CINAHL: Cumulative Index to Nursing & Allied Health
EMERGE: ESPACOMP Medication Adherence Reporting Guideline;
n: number
PEO: Population, Exposure, Outcome
PRISMA: Preferred Reporting Item for Systematic reviews and Meta-Analysis
